# A Guide for Developing Demo‐Genetic Models to Simulate Genetic Rescue

**DOI:** 10.1111/eva.70092

**Published:** 2025-05-14

**Authors:** Julian E. Beaman, Katie Gates, Frédérik Saltré, Carolyn J. Hogg, Katherine Belov, Kita Ashman, Karen Burke da Silva, Luciano B. Beheregaray, Corey J. A. Bradshaw

**Affiliations:** ^1^ Global Ecology | Partuyarta Ngadluku Wardli Kuu, College of Science and Engineering Flinders University Adelaide South Australia Australia; ^2^ Molecular Ecology Laboratory, College of Science and Engineering Flinders University Adelaide South Australia Australia; ^3^ Biogeography Ecology & Modelling, School of Life Sciences University Technology Sydney Sydney New South Wales Australia; ^4^ Australian Museum, Research Institute Australian Museum Sydney New South Wales Australia; ^5^ School of Life and Environmental Sciences The University of Sydney Sydney New South Wales Australia; ^6^ World Wide Fund for Nature Australia Melbourne Victoria Australia; ^7^ Conservation and Symbiosis Lab, College of Science and Engineering Flinders University Adelaide South Australia Australia

**Keywords:** conservation genetics, demography, density feedback, inbreeding depression, marsupials, *SLiM*, software

## Abstract

Genetic rescue is a conservation management strategy that reduces the negative effects of genetic drift and inbreeding in small and isolated populations. However, such populations might already be vulnerable to random fluctuations in growth rates (demographic stochasticity). Therefore, the success of genetic rescue depends not only on the genetic composition of the source and target populations but also on the emergent outcome of interacting demographic processes and other stochastic events. Developing predictive models that account for feedback between demographic and genetic processes (‘demo‐genetic feedback’) is therefore necessary to guide the implementation of genetic rescue to minimize the risk of extinction of threatened populations. Here, we explain how the mutual reinforcement of genetic drift, inbreeding, and demographic stochasticity increases extinction risk in small populations. We then describe how these processes can be modelled by parameterizing underlying mechanisms, including deleterious mutations with partial dominance and demographic rates with variances that increase as abundance declines. We combine our suggestions of model parameterization with a comparison of the relevant capability and flexibility of five open‐source programs designed for building genetically explicit, individual‐based simulations. Using one of the programs, we provide a heuristic model to demonstrate that simulated genetic rescue can delay extinction of small virtual populations that would otherwise be exposed to greater extinction risk due to demo‐genetic feedback. We then use a case study of threatened Australian marsupials to demonstrate that published genetic data can be used in one or all stages of model development and application, including parameterization, calibration, and validation. We highlight that genetic rescue can be simulated with either virtual or empirical sequence variation (or a hybrid approach) and suggest that model‐based decision‐making should be informed by ranking the sensitivity of predicted probability/time to extinction to variation in model parameters (e.g., translocation size, frequency, source populations) among different genetic‐rescue scenarios.

## 1 Introduction

Many threatened species only persist as small and isolated populations where the combined effects of inbreeding and genetic drift can accumulate deleterious mutations and reduce genetic diversity, together increasing the risk of extinction (Frankham 2005; Kardos et al. 2021). In addition to the protection and restoration of habitats, the successful conservation of many threatened species might require the deliberate movement of genetically differentiated individuals from one population to another to increase the genetic diversity and fitness of target populations—a process known as genetic rescue (Glossary—Table 1; Bell et al. 2019; Fitzpatrick et al. 2023; Ralls et al. 2020; Whiteley et al. 2015). However, genetic rescue is usually applied as an emergency intervention to prevent the extinction of small or declining populations that might already be vulnerable to random fluctuations in growth rates that can cause sudden population collapse (Melbourne and Hastings 2008; Bell et al. 2019; Fitzpatrick et al. 2023; Ralls et al. 2020; Whiteley et al. 2015). Therefore, the success of genetic rescue in conservation depends not only on the genetic composition and demographic history of the source and target populations but also on the emergent outcome of interacting demographic processes (and stochastic environmental events such as drought, fire, etc.). Hence, developing predictive models that account for feedback between demographic and genetic processes (‘demo‐genetic feedback’) is necessary to guide the implementation of genetic rescue to minimize the risk of extinction of threatened populations.

**TABLE 1:** Glossary. Relevant terms used in the main text with standard definitions from the ecology and evolution literature.

Term	Definition	References
Allele	Each of two or more alternative forms of a gene that arise by mutation and are found at the same place on a chromosome.	
Allee effect	Shift from compensation (negative relationship between per capita growth rate and abundance) to depensation (positive relationship between per capita growth rate and abundance) at small population sizes due to *inter alia* inbreeding depression, erosion of social networks, insufficient mating or rearing opportunities.	Courchamp et al. 1999; Herrando‐Pérez et al. 2012
Census population size (*N*_c_)	Number of individuals in a population, irrespective of their reproductive status or genetic contribution to future generations (compared to *effective population size*).	Frankham et al. 2019
Deleterious allele	Version of a gene that, on average, decreases fitness in the current environment of the individual.	
Demo‐genetic feedback	Reciprocal effects of demographic processes (e.g., density feedback, demographic stochasticity) on population genetic processes (e.g., genetic drift, selection, gene flow) that together determine population growth, genetic diversity and genetic load.	
Demographic stochasticity	Variance in population growth due to the chance nature of individual births, deaths, and migration.	Melbourne and Hastings 2008
Density feedback (~ density dependence)	When social and trophic interactions modify demographic rates and the resulting change in demographic rates alters population density, ‘feeding back’ to modify the intensity of those interactions.	Bradshaw and Herrando‐Pérez 2023
Dominance	Deviation of the phenotype of an individual that is heterozygous at a given locus from the mean phenotype of homozygous individuals. Complete dominance occurs when the heterozygous phenotype is indistinguishable from that of the homozygous parent. Partial (or incomplete) dominance occurs when the heterozygous phenotype is intermediate between the phenotypes of both homozygous parents (one of which is homozygous dominant and the other homozygous recessive).	Frankham et al. 2019
Drift load	Reduction in mean fitness due to stochastic increases in frequency of (usually weakly or moderately deleterious) mutations in small populations.	Lynch et al. 1995; Whitlock 2000
Epistasis	The dependency of the effects of gene substitutions on genetic background. Broadly speaking, interactions among genes at different loci (*c.f*. alleles at the same loci, see *dominance*). This definition refers to ‘functional epistasis’ (*sensu* Hansen 2013) *cf*. statistical epistasis (*sensu* Walsh and Lynch 2018).	Hansen 2013
Genetic load	Accumulation of deleterious mutations in a population. In terms of fitness, genetic load is the fraction by which the population mean differs from a reference genotype (i.e., the genotype with the maximum fitness). Mathematically, genetic load = realized load + masked load (= sum of selection coefficients of all deleterious mutations).	Dussex et al. 2023
Effective population size (*N*_e_)	The number of individuals that would result in the same loss of genetic diversity, inbreeding, genetic drift, or coalescence if they behaved in the manner of an idealized population. Notably, *N*_e_ is often smaller than the census population size due to factors such as unequal sex ratios, variation in reproductive success, and population fluctuations.	Frankham et al. 2019
Genetic rescue	Increase in population fitness (best measured by population growth rate) due to gene flow. Genetic rescue can occur due to natural or assisted gene flow. In the context of conservation of threatened species, genetic rescue can be more narrowly defined as deliberate genetic introductions aimed at masking deleterious alleles responsible for genetic load in small populations leading to an increase in population growth rate.	Whiteley et al. 2015; Hoffmann et al. 2021
Inbreeding depression	Reduced fitness of individuals with related parents. Distinct from *inbreeding* per se, which is the mating between individuals who are more closely related than the average randomly selected pair of individuals within a population.	Kardos et al. 2021
Locus, loci (pl.)	Position of a gene or a genetic marker on a chromosome.	
Recombination	Process in which pairs of chromosomes swap DNA with one another during gamete formation. Recombination brings new combinations of genes together—a source of variation upon which natural selection acts.	
Wright‐Fisher model	Discrete‐time, Markov chain model of the allele frequencies in a finite population of constant size, assuming random mating, non‐overlapping generations, no mutation, no migration, and no selection.	

The fields of ecology and population genetics each have their own rich set of deterministic (mean response) and stochastic models that have advanced predictions of the dynamics of natural populations and their genetic diversity, including elements of demo‐genetic feedback (Govaert et al. 2019; Nordstrom et al. 2023). But incorporating demo‐genetic feedback into applied models of genetic rescue is challenging because it typically requires approaches that are computationally expensive and necessarily complex (i.e., parameter‐rich and data‐hungry). Fortunately, rapid (and complementary) advances in computational power, sequencing technology, and genetically explicit, forward‐projection software are facilitating the development of sophisticated simulation models parameterized and validated with more data or based on more realistic assumptions than were previously possible (Haller and Messer 2019; Carey et al. 2019; Carturan et al. 2020; Pilowsky et al. 2022). These complementary advances enable the development and application of process‐explicit models needed to make more accurate predictions of the dynamics of wildlife populations under proposed management interventions such as genetic rescue.

Here, we explore the development and application of computational models to inform genetic rescue in conservation. We first summarize the theoretical and empirical literature on the demographic and genetic processes relevant to genetic rescue, and then suggest approaches for parameterizing models that incorporate demo‐genetic feedback mechanisms to improve predictions. We briefly outline the relative capabilities of five existing open‐source computer programs that could be used for genetic rescue simulations. Using one of these programs, we develop a heuristic model as a proof of concept for the influence of demo‐genetic feedback on simulated outcomes of genetic rescue. We then focus on Australian threatened marsupials as a case study and discuss how published genetic data could be used in model development and application (i.e., parameterization, calibration, and validation). We conclude by outlining a strategic direction for future research and application of model‐based decision‐making in genetic rescue.

## 2 Demo‐Genetic Feedback in Genetic Rescue

Genetic‐rescue interventions aim to reduce the probability of extinction of small and declining populations by countering genetic threats to fitness and population growth (Fitzpatrick et al. 2023). However, populations targeted for genetic rescue are usually vulnerable to a combination of stochastic genetic and demographic processes, the mutual reinforcement of which creates a positive feedback loop (demo‐genetic feedback) that heightens extinction risk as populations decline—a phenomenon referred to as the ‘extinction vortex’ (Caughley 1994; Fagan and Holmes 2006; Gilpin and Soulé 1986). Consequently, while genetic rescue can immediately improve fitness, these gains might be short‐lived if demographic instability persists, counteracting the benefits of genetic intervention and drawing the population back into the extinction vortex. Maximizing the effectiveness of genetic‐rescue interventions therefore requires a comprehensive understanding of demo‐genetic feedback and its role in shaping genetic‐rescue outcomes. In this section, we focus on the mechanisms of demo‐genetic feedback and highlight their relevance to genetic rescue. In the following section, we describe our suggested approach for building individual‐based simulation models of demo‐genetic feedback and how they can be applied to evaluate different scenarios of genetic‐rescue intervention.

**FIGURE 1:**
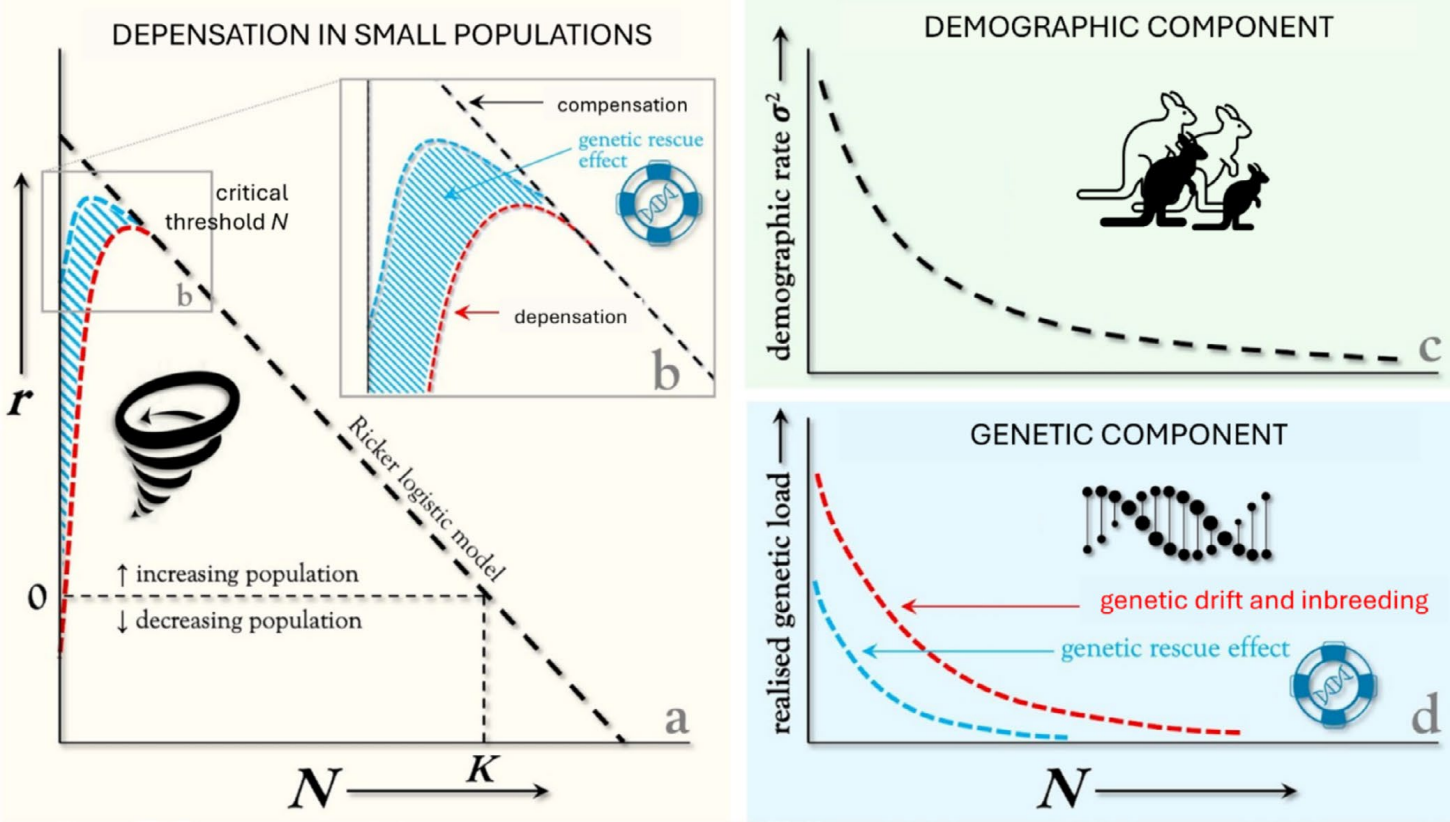
An example of demo‐genetic feedback in genetic rescue. (a, and inset b) Populations below a threshold abundance (*N*) often exhibit decreasing per‐capita population growth rates (*r*) at lower *N*, which is a type of density dependence known as depensation. This contrasts with the compensation that is typical of density feedback at larger population sizes (represented by the Ricker logistic model of linear decline in *r* with increasing *N*). The dotted vertical line denoted by *K* represents the population size at carrying capacity, which is mathematically defined as the long‐term mean population density (or size, *N*) where *r* = 0. (b, c) Several mechanisms cause depensation, including (c) demographic stochasticity (random fluctuations in population growth rate or abundance) due to increased variance in demographic rates (e.g., survival, fertility) at small population sizes, and (d) genetic effects due to increased genetic drift and inbreeding at small population sizes, which increase the realized genetic load and reduce mean fitness. The mutually reinforcing interaction between demographic and genetic effects increases extinction probability in small populations (i.e., the extinction vortex). Ecological and behavioral mechanisms of depensation (e.g., mate limitation, social structure disruptions) that give rise to what is also referred to as the Allee effect (Courchamp et al. 1999) are not shown to retain a focus on demo‐genetic‐feedback mechanisms. Genetic drift and inbreeding can also be considered akin to genetic mechanisms of the Allee effect (Luque et al. 2016). It is these genetic mechanisms of population depensation that genetic rescue specifically seeks to reduce.

Small populations are strongly influenced by stochastic processes that emerge at the population level from underlying individual‐level and genetic mechanisms. The discrete and binary nature of birth and death events introduces randomness into demographic rates (e.g., survival and fertility) and emergent phenomena (e.g., sex ratios, age of reproductive maturity), causing stochastic fluctuations in population growth rate and abundance (demographic stochasticity). Fluctuations in abundance pose little threat to larger populations because declines in density tend to improve mean fitness (e.g., through reduced competition), leading to compensatory increases in per‐capita population growth rate (compensation, Herrando‐Pérez et al. 2012). In smaller populations, however, demographic stochasticity can cause declines in abundance below a threshold where they become *depensatory*—when subsequent declines in density decrease mean fitness and engender further decline (Figure 1). Depensatory population decline can be caused by ecological and behavioral mechanisms (e.g., mate limitation, social structure disruptions), phenomena known to give rise to what is also referred to as the Allee effect (Courchamp et al. 1999). Depensation can also be caused by genetic mechanisms (e.g., genetic drift and inbreeding) that can be considered akin to genetic mechanisms underlying the Allee effect (Luque et al. 2016). It is these genetic mechanisms of population depensation that genetic rescue specifically seeks to offset.

As with stochastic fluctuations in demographic rates, the discrete and binary nature of individual birth and death underpins the random genetic drift of allele frequencies in populations. Due to the strong influence of genetic drift in small populations, weakly or moderately deleterious mutations can accumulate and become fixed, thereby reducing mean population fitness (drift load; Angst et al. 2022; Whitlock 2000). Inbreeding also becomes more prevalent as populations decline, and increases the likelihood that recessive deleterious mutations will become expressed in homozygous individuals to reduce their fitness (i.e., inbreeding depression) (Charlesworth and Willis 2009; Frankham 2005; Frankham et al. 2019; O'Grady et al. 2006). By increasing the frequency of deleterious alleles and reducing genetic diversity, genetic drift and inbreeding therefore convert masked genetic load that was previously hidden in the heterozygous state into realized genetic load, ultimately reducing population growth and resilience (Dussex et al. 2023; Mathur and DeWoody 2021). Strongly deleterious mutations are less likely to accumulate through genetic drift because their contribution to inbreeding depression tends to promote their removal by natural selection (genetic purging), even in small populations (Dussex et al. 2023; Grossen et al. 2020; Hedrick and Garcia‐Dorado 2016). The role of strongly deleterious mutations and genetic purging in the success of genetic rescue is a current and contentious topic of debate (Kyriazis et al. 2021; Pérez‐Pereira et al. 2022; Ralls et al. 2020; Robinson et al. 2018; Robinson et al. 2021), and is a research area in which simulation models have the potential to make a valuable contribution.

To reduce the threat that realized genetic load poses to small and declining populations, genetic‐rescue interventions deliberately introduce heterozygous and genetically differentiated individuals into a targeted population in an attempt to remask deleterious alleles contributing to genetic load, thereby eliciting an increase in population growth rate (see Hoffmann et al. 2021 for a review of different forms of genetic mixing in conservation). The success of genetic rescue hinges on whether masking genetic load increases mean fitness enough to increase population abundance above the threshold where population growth switches from depensation to compensation (Figure 1). Individual‐based models provide a powerful and practical tool with which to compare and rank alternative scenarios of genetic‐rescue interventions based on the outcomes of virtual genetic rescue in simulated populations. Models that incorporate the demo‐genetic feedback mechanisms driving depensatory dynamics can be used by conservation practitioners to make forward projections of the impact of introducing genetically differentiated (virtual) individuals on realized genetic load and growth rate of (simulated) populations. In the following section, we suggest approaches for developing demo‐genetic models with which to simulate genetic rescue, and provide a brief overview of open‐source software and its relevant capabilities.

## 3 Building Demo‐Genetic Simulations of Genetic Rescue

### 3.1 Linking Feedback Mechanisms to Model Parameters

Computational tools have advanced to where it is now possible to develop practical simulations to plan and implement scenarios of genetic rescue. Genetically explicit, individual‐based models are the most promising for incorporating demo‐genetic feedback mechanisms to evaluate the probability of success of genetic rescue of small, isolated populations. Open‐source software programs capable of simulating the influence of demo‐genetic feedback on the outcome of genetic rescue include *SLiM* (Haller and Messer 2023), *quantiNemo* (Neuenschwander et al. 2019), an extension of the original *Nemo* software (Guillaume and Rougemont 2006), *CDMetaPOP* (Day et al. 2023), *RangeShifter* (Bocedi et al. 2021), and *HexSim* (Schumaker and Brookes 2018). While there are likely other custom models, these five platforms contain most of the processes required to integrate demo‐genetic processes in genetic‐rescue scenarios. Here, we start by describing the types of parameters that could be specified in genetic rescue simulations to permit the emergence of demo‐genetic feedback from underlying mechanisms (Table 2). We then provide a brief description of each of these five platforms and summarize their capabilities for parameterizing the mechanisms of demo‐genetic feedback (Table 3).

**TABLE 2:** Parameterizing demo‐genetic feedback in genetic‐rescue simulations. We provide examples of the types of parameters that could be used to model the emergence of demo‐genetic feedback and depensatory dynamics in genetically explicit, individual‐based simulation models. We focus on the component mechanisms of demo‐genetic feedback, for each of which we describe the relevant aspect of its relationship with population abundance and suggest model parameters linked to underlying mechanisms used to simulate its emergence in virtual populations. Figures 1 and 2 provide visual guides to the relationships among the processes and mechanisms described in the table.

Component mechanism	Feedback with abundance	Effect on fitness	Relevant model parameters
Demographic stochasticity (random variance in population growth rate)	Increasing variance in component demographic rates (survival, fertility) as abundance decreases	Independent of fitness	Demographic rates: specifically, parameters used to cause increasing variance in demographic rates as abundance declines (see Supporting Information)
Genetic drift	Increased influence of genetic drift in small populations	Causes accumulation and fixation of deleterious alleles	Population allele frequencies: either generated via virtual *de novo* mutation during simulation burn‐in, or user‐specified allele frequencies or sequence data at model initialization Recombination rate(s) Allelic dominance coefficients for deleterious mutations/alleles Fitness effects of deleterious mutations/alleles
Inbreeding	Increased incidence of inbreeding as population abundance decreases	Reduces individual fitness due to homozygosity at deleterious loci (inbreeding depression); Reduces mean fitness due to homozygosity at deleterious loci (realized genetic load)	*Incidence* of inbreeding in virtual populations an emergent process in individual‐based models that include sexual reproduction *Outcome* of inbreeding on prevalence of inbreeding depression and realized genetic load mediated by allele frequencies, recombination rates, dominance, and fitness effects
Genetic purging	Efficacy of selection in purging deleterious alleles usually weaker in smaller populations (due to increased influence of drift); inbreeding in small populations can also increase efficacy of purging	Increases mean fitness due to removal of deleterious alleles from the population	Allele frequencies Recombination rates Allelic dominance Fitness effects

**TABLE 3:** Comparison of the capability and flexibility of existing open‐source software for building demo‐genetic simulations of genetic rescue. We emphasize that the comparisons provided here are specific to use cases we describe in the main text and do not reflect a comparison of the programs for other use cases. For each broad capability category (left‐hand column), we specify different levels of capability in each row in the column labeled ‘flexibility’. For each program, we indicate whether it does or does not possess a given level of capability. Where a program is flexible enough to be able to perform a higher level of capability, we use a dash (—) to indicate that it can also perform a lower‐level capability. Where we were unable to ascertain whether a program had a given level of capability (from publicly available material such as user manuals and journal articles), we left that element blank. Footnotes for superscript symbols (numbered in order of appearance within each row) are provided at the bottom of the table. The interpretations of other symbols used in the table are: $f\left(\sigma_{D}^{2},N\right)$ represents the capability to model increasing *random variance* in demographic rates ($\sigma_{D}^{2}$—as distinct from environmental or genetic sources of variance) as a function of decreasing population abundance (*N*); *r*($\sigma_{D}^{2}$, *N*) represents the correlation between the random variance in demographic rates ($\sigma_{D}^{2}$) and mean demographic rates ($\mu_{D}$), with programs differing in their flexibility to model $\sigma_{D}^{2}$ independently of $\mu_{D}$ i.e. *r*($\sigma_{D}^{2}$, $\mu_{D}$) = 0.

Capability	Flexibility	*SLiM*	*quantiNemo* ^a^	*CDMetaPop*	*RangeShifter*	*HexSim*
Genetics						
*De novo* mutations	Single rate	Yes	Yes	Yes	Yes	Yes
	Multiple rates	Yes	Yes	Yes	No	Yes
Virtual alleles	Abstract^b^	Yes	Yes	Yes	Yes	Yes
	Mapped sequences^c^	Yes^d^	Yes^e^	No	No	Yes^f^
Empirical alleles	Genotype‐informed	—	—	Yes	Yes	Yes
	Genotype file input	Yes^g^	Yes^h^	Yes	No	No
	Mapped sequences	Yes^i^	Yes^j^	No	No	No
Fitness effects of mutations	Relative^k^	Yes	Yes	Yes^l^	Yes^m^	
	Absolute^n^	Yes	Yes	No	No	
	Point values	—	—	Yes	—	
	Distributional (fixed)	—	—	No	Yes	
	Distributional (User‐specified)	Yes	Yes	No	No	
Recombination	Crossover	Yes	Yes	No	Yes	Yes
	Non‐crossover	Yes	Yes	No	No	No
	Single rate	—	—	No	Yes	
	Multiple rates	Yes	Yes	No	No	
(Allelic) dominance		Yes	Yes	No	No	No
Demography						
Source of *variance* in individual survival probability	Environment	Yes	Yes	Yes	Yes	Yes
	Genotype	Yes	Yes	Yes	No	
	Random	Yes	No	Yes	Yes	
	Stage‐specific	Yes	No	Yes	Yes	Yes
Source of variance in individual fertility rate	Environment	Yes	Yes	Yes	Yes	Yes
	Genotypic effects	Yes	Yes	Yes	No^o^	
	Random	Yes	Yes	Yes	Yes	
*f*($\sigma_{D}^{2}$, *N*)	*r*($\sigma_{D}^{2}$, $\mu_{D}$) ≠ 0	—	No	No	Yes	No
	*r*($\sigma_{D}^{2}$, $\mu_{D}$) = 0	Yes	No	No	No	No
Stage‐specific demographic rates	Survival	Yes	Yes	Yes	Yes	Yes
	Fertility	Yes	No	Yes	Yes	Yes
Sex ratio	Fixed	—	—	—	—	
	User‐defined	Yes	Yes	Yes	Yes	
Generations	Non‐overlapping	—	Yes	Yes	Yes	—
	Overlapping	Yes	No	Yes	Yes	Yes
Spatiality	Habitat suitability	Yes	Yes	Yes	Yes	Yes
	Landscape permeability	Yes	Yes	Yes	Yes	Yes
Dispersal	Kernel‐based	Yes	Yes	Yes	Yes	Yes
	Path‐based	No	No	Yes	Yes	Yes

^a^ Extension of *Nemo* and related to *Nemo‐age*.

^b^ Virtual alleles are described as abstract if they have properties such as selection coefficients or position in the genome, but do not explicitly represent a (virtual) nucleotide sequence.

^c^ Virtual alleles are specified with some form of physical location.

^d^ Virtual genome.

^e^ Recombination map.

^f^ Unitless type of virtual linkage map.

^g^ VCF file.

^h^ FSTAT file.

^i^ FASTA file.

^j^ Arlequin file.

^k^ Fitness varies relative to environmental context and/or other individuals.

^l^ Relative fitness effects implemented via fitness landscapes for diallelic (single‐locus or two‐loci) models.

^m^ Fitness effects implemented via survival probability during three stages of dispersal (emigration, transfer, settlement) and density‐ and habitat‐specific effects on survival and fertility rates.

^n^ Useful for modelling genetic load.

^o^ Although variance in dispersal traits, which are heritable, indirectly influences variation in fertility rate via density‐dependent fecundity, which varies spatially.

**FIGURE 2:**
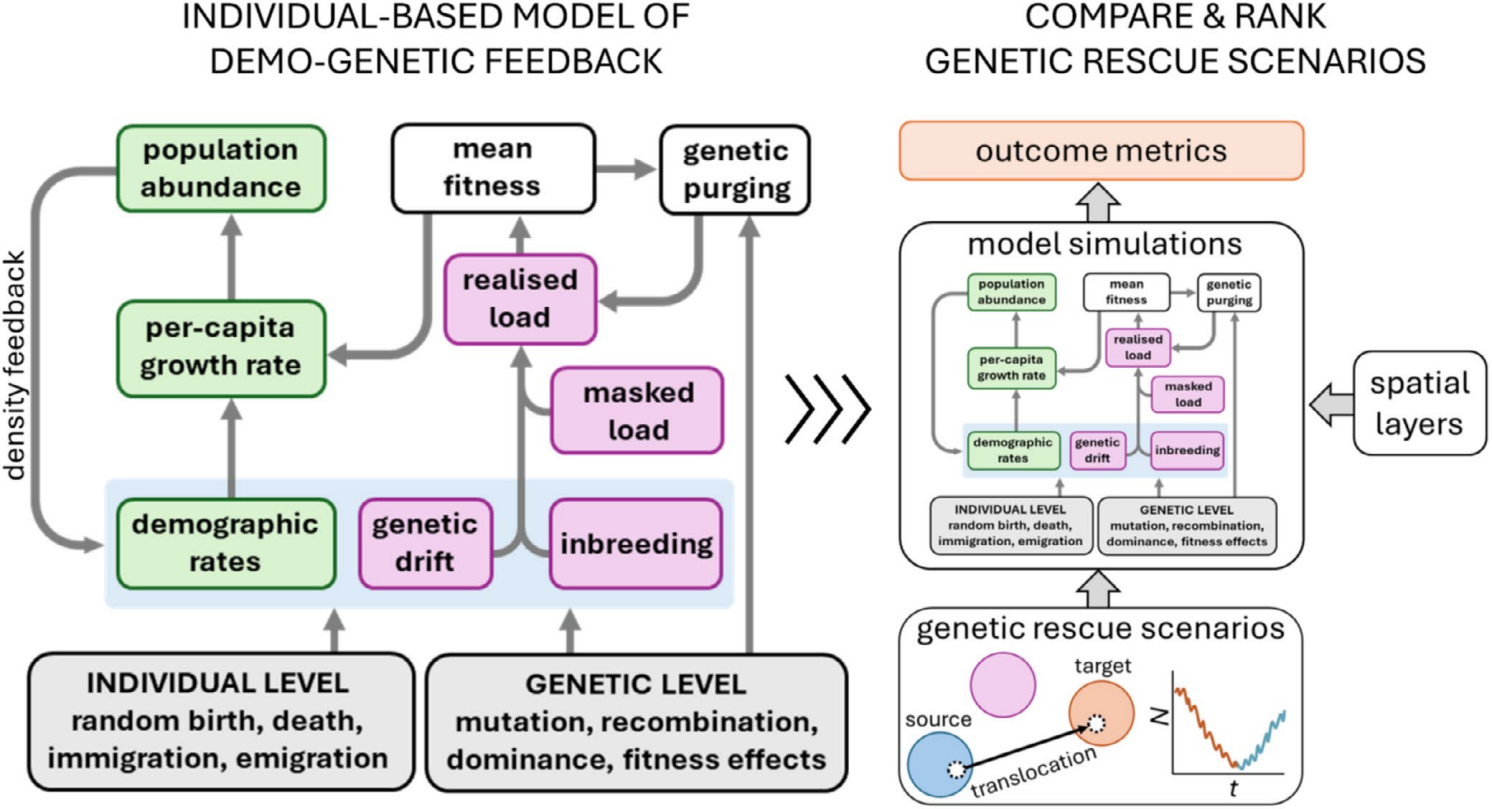
Individual‐based models of demo‐genetic feedback and simulations of applied genetic‐rescue scenarios. Left panel: The discrete and random nature of individual‐level and genetic mechanisms causing demographic and genetic stochasticity to emerge at the population scale (blue‐shaded box). Stochastic processes cause fluctuations in per‐capita population growth rate and the frequency and expression of deleterious alleles. Mutual reinforcement of demographic and genetic processes is driven by the effect of mean fitness on per‐capita growth rate and density feedback, as well as the strength of demographic and genetic processes (within the blue‐shaded box) on per‐capita growth rate and realized genetic load. We suggest how to link individual‐level and genetic mechanisms to model parameters in Table 2. We provide the capability and flexibility of available software for specifying parameters in Table 3. Right panel: Once the individual‐based, demo‐genetic model is parameterized and calibrated, it can be used to simulate the outcome of alternative scenarios of (virtual) genetic‐rescue interventions. Scenarios can be compared and ranked according to their relative success in decreasing mean time to extinction (*T*_*ext*_) and the probability of extinction (*Pr*(*extinct*)), at time *t* (‘outcome’ metrics). We provide a guide to the application of model simulations to inform genetic rescue in section *Using simulations to inform applied genetic rescue*.

Individual‐based models for simulating genetic rescue have the advantage of replicating the discrete and binary nature of births, deaths, immigration, and emigration. This introduces randomness (i.e., variance) into probabilities of survival and fertility, and other emergent demographic properties such as sex ratio and age structure, that underpin stochastic fluctuations in per‐capita growth rate (*r*) and abundance (*N*) (Figure 2). Similarly, genetically explicit, individual‐based models can simulate the stochastic accumulation of deleterious alleles via genetic drift, and their contribution to inbreeding depression and realized genetic load (although modelling platforms differ in the specific details of how these processes are represented, described below in the section on software capabilities). For example, stochastic genetic processes can be modeled by setting parameters for mutation rates (or initialized allele frequencies), recombination rates, allelic dominance, and the fitness effects of mutations or alleles (either drawn from probability distributions or fixed), including neutral, beneficial, or deleterious effects. There are limits to which of these parameters can be calibrated and validated with empirical data, and we address this topic below using a case study of threatened Australian marsupials in *Using data to guide model development and application*.

Available software programs are capable of modelling a wide range of ecological and evolutionary processes. Therefore, simulating genetic rescue scenarios to inform specific conservation actions requires making important decisions about which parameters to include or exclude for developing a parsimonious model capturing the essential dynamics of the system (see García‐Díaz et al. 2019 for a concise guide to the development and use of quantitative models in conservation management). We contend that the implementation of genetic rescue in conservation should be guided by models that replicate the *mutual reinforcement* of demographic stochasticity, genetic drift, and inbreeding (e.g., Figure 2). These processes cause populations to decline into the extinction vortex (Figure 1) and create uncertainty for decision‐makers choosing among a set of competing potential interventions.

### 3.2 Incorporating Spatial and Environmental Dynamics

Most populations exhibit spatial variation in both demographic rates and genomic properties (White and Smith 2018), so spatially explicit models of genetic rescue are likely to be more realistic than non‐spatial models. Furthermore, individuals that are translocated from source population(s) into the target population might tend to hyper‐disperse from the release site (Bilby and Moseby 2024), with implications for subsequent genetic mixing. Spatial models can account for dispersal dynamics (e.g., dispersal propensity and distance), which might be influenced by spatial variation in habitat suitability and configuration, as well as permeability (or resistance) to movement.

### 3.3 Capabilities of Existing Open‐Source Software for Building Demo‐Genetic Simulations of Genetic Rescue

Here we provide a brief description of five open‐source software programs that could be used to develop and run demo‐genetic simulations of genetic rescue. Each program has detailed documentation and support materials useful for applied researchers and conservation practitioners. The written summary of each program is intended to provide context rather than a detailed comparison of their capability and flexibility for developing demo‐genetic models for simulating genetic rescue, which we provide in Table 3. There, we identify specific types of parameters relating to the mechanisms and processes we describe in sections above (i.e., parameters that give rise to the emergence of demo‐genetic feedback in virtual populations). For each program, we state whether it does or does not have the capability or flexibility we describe for each parameter type. For example, we differentiated among the programs based on whether they model virtual mutations/alleles as ‘abstract’ variants or ‘mapped sequences’ (or both). We categorized mutations/alleles as abstract if they are treated as variants defined by a set of properties (e.g., selection and dominance coefficients), which do not include a physical location (e.g., in a virtual genome or recombination map). Conversely, we categorized mutations/alleles as mapped sequences if they were treated as nucleotide variants with a defined location on a virtual genome or recombination map. This is one example of the approach we take to comparing the programs in Table 3. We start below with a brief descriptive summary of each program.

*SLiM* (‘**S**election on **Li**nked **M**utations’; messerlab.org/slim) (Haller and Messer 2023) is an individual‐based, forward‐in‐time simulator designed for studies of evolutionary genetics. *SLiM* is genetically explicit in that it simulates mutations at specific positions along a virtual genome rather than allele frequencies or quantitative genetic trait means and variances. By default, *SLiM* models the genome as a ‘blank slate’ of chromosomal positions and only mutations are tracked to increase computational efficiency. However, it is possible to simulate empirical nucleotide sequences in *SLiM* using FASTA (a text‐based format for representing nucleotide sequences as single‐letter codes) and VCF (variant call format, a standard text file for storing gene‐sequence variation) files to represent the simulated sequences. Other genetic processes that can be modelled include variable recombination rates across the genome (emulating genetic linkage and discrete chromosomes) and variation in allelic dominance. The latter allows the modelling of partially recessive deleterious alleles, which are important contributors to genetic load (Charlesworth and Willis 2009; Hedrick and Garcia‐Dorado 2016). Epistatic interactions (when gene expression is modified by the expression of ≥ 1 other genes) can also be modelled in *SLiM* and simulations offer a powerful tool with which to understand the influence of epistasis on the accumulation, expression, and purging of deleterious mutations in small populations. However, the role of epistasis in the extinction and genetic rescue of small populations is beyond the scope of the current paper. Recent versions of *SLiM* (currently V4.3) relax the assumptions of classical Wright‐Fisher models (see Glossary) of population genetics and can simulate a range of ecologically realistic processes, including overlapping generations, age structure, density feedback on population growth, variation in individual fertility and survival, as well as species interactions useful for modelling predator–prey or infectious disease dynamics (Haller and Messer 2019, 2023). *SLiM* models can be spatially explicit, with individuals modelled in continuous space (up to 3 dimensions), and gridded layers of environmental variables.

*quantiNemo* (Neuenschwander et al. 2019) is an extension of the original *Nemo* program (Guillaume and Rougemont 2006; nemo2.sourceforge.io), which has also been extended as *Nemo‐age* (Cotto et al. 2020). Here, we focus on *quantiNemo* because it has expanded capacity relevant for simulating genetic rescue. *quantiNemo* is an individual‐based, genetically explicit stochastic simulation program. It was developed to investigate the effects of selection, mutation, recombination, and drift on quantitative traits with varying architectures in structured populations connected by migration and located in a heterogeneous habitat. *quantiNemo* is flexible in several components: population, selection, trait(s) architecture, genetic map for quantitative trait loci and/or markers, environment, demography, mating system, etc. It is an object‐oriented console program coded in C++, runs on any computer platform, and is distributed under an open‐source licence. In contrast to *SLiM*, which models mutations on a virtual chromosome, *quantiNemo* implements a recombination map on which loci coding for different types of traits can be placed together. The genetic processes (and evolution of quantitative traits) modelled in *quantiNemo* relevant to simulations of genetic rescue include deleterious mutations, neutral markers (microsatellites, single nucleotide polymorphisms), quantitative trait loci (QTL), sex‐specific dispersal rates, and life‐history traits, among others. The framework supports spatial structure, as well as various genetic architectures useful for simulating genetic drift, gene flow, and selection. Unlike *SLiM* and *CDMetaPop* (described below), *quantiNemo* can only model single species, but the number of simulated loci, individuals, or populations is only limited by computational power. We do not know of any studies that have compared the computational efficiency of *quantiNemo* models of varying complexity. Hence, we cannot provide an indication of a realistic upper limit to these parameters, which would need to be optimised on a case‐by‐case basis.

*CDMetaPOP* (‘**C**ost‐**D**istance **M**eta‐**POP**ulation’; github.com/ComputationalEcologyLab/CDMetaPOP) (Day et al. 2023; Landguth et al. 2017) is a simulation program used to predict the influences of landscape structure and individual‐based movement, breeding, and dispersal on the emergence of spatial patterns in population genetic data (i.e., landscape genetics). Like *quantiNemo*, *CDMetaPop* treats the landscape as a lattice of patches within which individuals share a common environment, with the option of specifying patches at the individual scale. Specifying among‐patch environmental variables creates a heterogeneous landscape, with movement of individuals across this landscape determined by resistance (or permeability) surfaces. Within patches, individuals grow, reproduce, migrate, and die. These ecological processes give rise to spatially explicit neutral or adaptive changes in allele frequencies at an unlimited number of loci and alleles. Unlike *SLiM* (genotypes as virtual genomes) and *quantiNemo* (genotypes as recombination maps), individual genotypes in *CDMetaPop* comprise lists of user‐specified loci and alleles. The model is initialized with population‐level allele frequencies (either hypothetical values or empirical estimates) and offspring inherit genotypes based on Mendelian inheritance. Users can choose from a number of mutation models (e.g., *k*‐allele, step‐wise). Single nucleotide polymorphisms can be accommodated in the model as bi‐allelic loci. Demographic rates (e.g., birth, death, dispersal) are specified as probabilities, with individual outcomes allocated binomially, meaning that demographic stochasticity is implicit. Environmental variables can vary across both space and time, and specifying a mean and variance incorporates environmental stochasticity. Like *SLiM*, the most recent incarnation (*CDMetaPop* V2) expands the capacity to model eco‐evolutionary dynamics in multi‐species communities. The numbers of loci, alleles, individuals, and species that can be modeled are limited only by computational power, although it is recommended that loci × alleles < 10^5^ (Day et al. 2023; Landguth et al. 2017). We do not know of any studies that have compared the computational efficiency of *CDMetaPop* models of varying complexity. Hence, we cannot provide an indication of a realistic upper limit for all combinations of parameters, which would need to be optimized on a case‐by‐case basis.

*RangeShifter* (rangeshifter.github.io) (Bocedi et al. 2021) is a modelling tool designed for simulating the ecological and evolutionary dynamics of individual species in response to environmental shifts. The platform incorporates temporally varying landscapes, explicit genetic modelling, and the evolution of dispersal mechanisms with a particular focus on identifying population risks and understanding processes driving species range dynamics, including the impact of landscape structure on genetic diversity and adaptation. The genetic aspect of *RangeShifter* offers detailed modelling of heritable traits, supporting the evolution of dispersal strategies and providing a basis for advanced studies of landscape genetics. While *RangeShifter* offers robust insight into species' responses to environmental changes, the utility of the genetic aspect is tempered by the absence of certain functionalities, like the nuanced representation of genetic processes found in other software such as *SLiM, quantiNemo*, and *CDMetaPop*. *RangeShifter* also lacks the capacity to model interspecific interactions like *SLiM* and *CDMetaPop*. Because *RangeShifter* provides a new framework for dispersal modelling and connecting movement ecology with spatial dynamics, it is still a potentially useful tool for simulating genetic rescue.

*HexSim* (hexsim.net) (Schumaker and Brookes 2018) is a spatially explicit, individual‐based model designed for studying wildlife population dynamics across diverse landscapes and scenarios. It focuses on spatial complexity that influences individual behaviours such as movement, survival, reproduction, and dispersal, thereby affecting overall population dynamics and distributions. Not only can *HexSim* simulate complex population dynamics, including fluctuations in population sizes, age structure, and the effects of environmental perturbations, it also incorporates genetic factors to examine gene flow, genetic diversity, and the effects of landscape on genetic structure. These genetic components can include tracking alleles or genotypes over time, assessing the effects of drift and selection, and evaluating the impacts of landscape connectivity on genetic diversity. The model links life‐history traits with demographic and genetic components, allowing for the simulation of selective pressures on genetic traits and their demographic consequences. *HexSim* integrates demographic processes with spatial landscape features to offer insights into population viability, genetic health, and conservation requirements.

We acknowledge that there are other individual‐based, genetically explicit software programs that might be equally applicable to modelling genetic rescue. For example, *SimAdapt* (Rebaudo et al. 2013) based on the *NetLogo* modelling environment (Wilensky 1999) and *Geonomics* (Hart et al. 2021) are two examples, and there are likely others. Users might differ in what elements of a program they find more useful in each context. It is beyond the scope of our paper to provide a thorough review of the capabilities of all the available programs. Instead, our focus here is to compare the capabilities of a representative set of five programs to support our main message—that the implementation of genetic rescue in conservation should be guided by models that replicate the mutual reinforcement of demographic stochasticity, genetic drift, and inbreeding.

## 4 Example Demo‐Genetic Model for Simulating Genetic Rescue

### 4.1 Model Development

**FIGURE 3:**
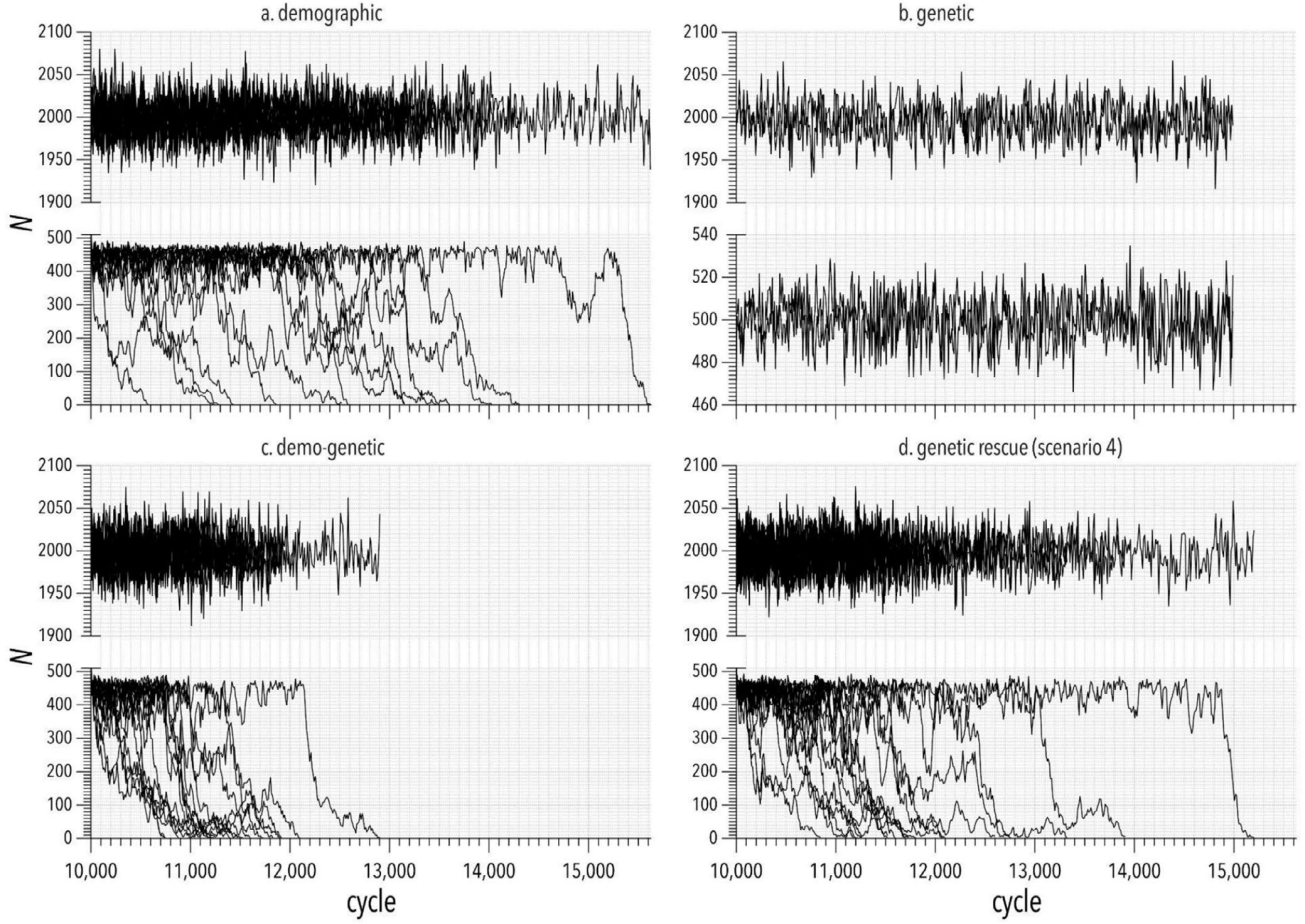
Fluctuations in population abundance. Example simulations of different models that demonstrate the influence of (a) demographic stochasticity, (b) genetic effects, (c) demo‐genetic feedback, and (d) genetic rescue (scenario 4; see description in text) on abundance (*N*) over time. Each panel (a–d) shows two time series that were both run in parallel for a burn‐in of 10,000 simulation cycles (≈ years) and both reach an equilibrium *N* that fluctuated around the carrying capacity *K*_1_ of 2000 individuals. In each panel, the time series at the top represents the control population with *N* maintained at *K*_1_. The time series at the bottom of each panel represents the focal population that experienced an abrupt crash in *N* at cycle 10,001 to a new *K*_2_ = 500. Each panel shows a representative sample of up to 17 iterations of the simulation scenario (*n* = 120 per scenario in model results in main text). Each iteration shows a different trajectory of population abundance over time until extinction (at which point we stopped both control and focal population iterations and restarted them in the next iteration). The effect of demo‐genetic feedback on extinction probability is illustrated by comparing the bottom time series of (c) to that of (a) and (b). The effect of genetic rescue is illustrated by comparing the bottom time series of (c) with that of (d). Scale of the *y* axes differs between upper and lower plots in each panel.

Here, we develop simulations of a heuristic model to demonstrate the influence of demo‐genetic feedback on extinction dynamics and the outcomes of genetic rescue of small populations. We modelled a hypothetical population that has suffered an abrupt crash in population size. The life history of our hypothetical species is loosely inspired by koalas ( *Phascolarctos cinereus* ), but the specific parameters we used were not empirically informed or calibrated because our goal is to demonstrate one example of how to build a model in which demo‐genetic feedback emerges from underlying individual‐level and genetic mechanisms (Figure 2) and influences the outcome of virtual genetic rescue. We start by developing a set of three base models in which simulated population dynamics emerge from demographic stochasticity only (demographic model), genetic drift and inbreeding only (genetic model), and the mutual reinforcement of demographic stochasticity, genetic drift, and inbreeding (demo‐genetic model) (Figure 3; see Supporting Information for a detailed description of how we constructed the models).

We used *SLiM* (*v*4.0; Haller and Messer 2023) to build the models. For each of the three models, we simulated two hypothetical populations—one that maintained an equilibrium population size at a carrying capacity (*K*_1_) of 2000 individuals (P_1_ = control population) for the entire simulation, and another that experienced an abrupt demographic decline (e.g., due to habitat loss) to a carrying capacity (*K*_2_) of 500 individuals (P_2_; focal population). The abrupt decline occurred after a burn‐in period of 10,000 cycles (replicated in each iteration), which approximately equated to years in the model (see *Temporal resolution of simulation cycles and iterations*). Following the abrupt decline in abundance in the focal population, we ran each iteration of the simulation until the population went extinct. For each iteration, we recorded the time to extinction *T*_*ext*_ measured in simulation cycles (≈ years), from which we calculated mean *T*_*ext*_ across all iterations for a given model. For each model, we also calculated an extinction probability *Pr*(*ext*) calculated as 1 minus the fraction of iterations in which the focal population remained extant after an arbitrary time of 1500 cycles (≈ years) after the population declined. We used *T*_*ext*_ and *Pr*(*ext*) to compare the influence of demographic stochasticity, drift and inbreeding, and demo‐genetic feedback, respectively, on population viability.

Next, we used the base demo‐genetic model to examine the effect of different genetic‐rescue scenarios on *T*_*ext*_ and *Pr*(*ext*). We emphasize that this exercise is intended as a proof‐of‐concept and not to guide management decisions. We used the demo‐genetic model to examine the outcome of four main genetic‐rescue scenarios on *T*_*ext*_ and *Pr*(*ext*). In each scenario, we varied: (*i*) the number of individuals moved from the control population (P_1_ with K_1_ = 2000) into the focal population (P_2_ with K_2_ = 500) in any single ‘translocation’ event (cohort size), and (*ii*) the number of translocation events. The four scenarios were: **Scenario 1**: 50 individuals moved once at 250 years after demographic decline; **Scenario 2**: 100 individuals moved once at 250 years after demographic decline; **Scenario 3**: 50 individuals moved three times at 250, 255, and 260 years after demographic decline; and **Scenario 4**: 100 individuals moved three times at 250, 255, and 260 years after demographic decline.

In the four scenarios, we considered only the effects of cohort size and the number of translocation events. There are a range of other variables that decision‐makers might consider when implementing genetic rescue in natural populations. Below, in *Using simulations to inform applied genetic rescue*, we provide a detailed discussion of how simulations can be used to compare and rank genetic‐rescue scenarios that differ in a wide range of variables. Here, it suffices to focus on factors that commonly vary among implementations of genetic rescue. All *SLiM* code required to run the simulations is available at https://zenodo.org/doi/10.5281/zenodo.10939288.

### 4.2 Heuristic Model Simulation Results

**FIGURE 4:**
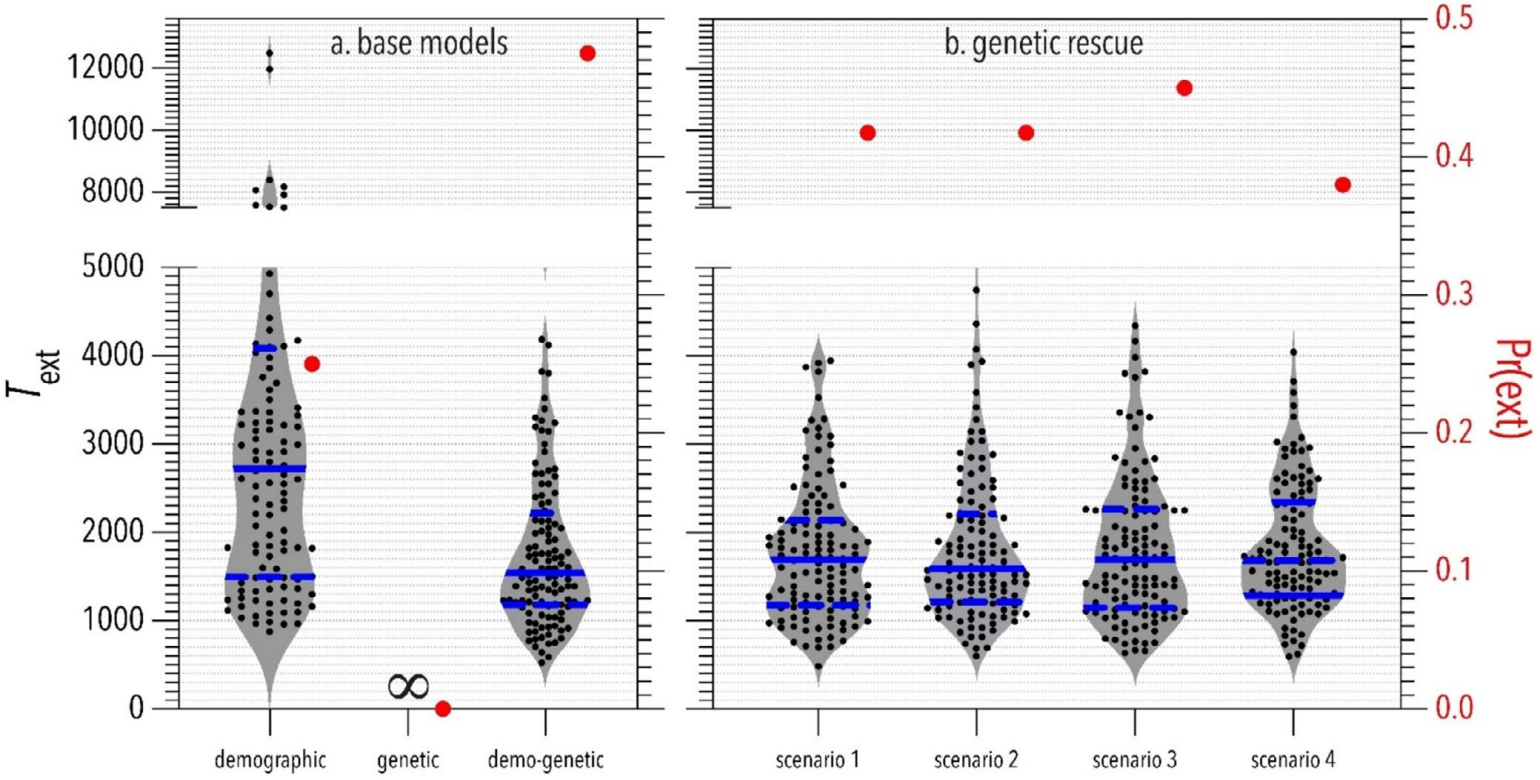
Comparing the outcomes of (a) models that incorporate different component effects, and (b) four genetic‐rescue scenarios (see description in text) based on the demo‐genetic model on population viability. Grey‐shaded violin plots (with black dots indicating individual simulations) show time to extinction (*T*_ext_, left *y* axis) measured in simulation cycles (≈ years) starting from the abrupt demographic decline of the focal population from a carrying capacity (*K*_1_) = 2000 during the burn‐in period to *K*_2_ = 500 individuals. Solid horizontal blue lines show median *T*_ext_, and dashed blue lines show quartiles. Red dots show extinction probability (right *y* axis), Pr(ext) = 1 minus the proportion of iterations where the focal population was extant at 1500 years (simulation cycles) after the abundance decline. The control populations (*K*_*1*_) in all scenarios had no extinctions and 100% persistence probability. ∞ = no extinction. The main outcomes to note are: (*i*) simulations of the demo‐genetic base model (a) had higher extinction probability (red dots) and shorter time to extinction (violin plots) than base models that only incorporated demographic or genetic components, respectively; and (*ii*) simulated genetic rescue reduced extinction probability by 3%–9% relative to the demo‐genetic base model, with scenario 4 having the greatest reduction.

In the demographic model, variance in survival probability caused abundance to fluctuate randomly over time (Figure 3) and gave an extinction probability *Pr*(*ext*) = 0.24 for the focal population (Figure 4). In the genetic model, deleterious mutations that were partially recessive (i.e., alleles with incomplete dominance; Glossary, Table 1) accumulated (at low frequency) in both the focal and control populations, but a reduction in abundance alone was not enough to cause inbreeding and genetic drift to overwhelm compensatory dynamics (i.e., population growth towards carrying capacity, *K* = 500) to unmask the genetic load and reduce mean fitness and population growth rate (Figure 3). In other words, without stochasticity in survival, deleterious mutations were purged through selection acting on individuals that were homozygous for deleterious mutations (inbreeding depression). Hence, without the additional effects of demographic stochasticity (and with *K* = 500), there were no extinctions in simulations of the genetic model (Figure 4).

In the demo‐genetic model, demographic stochasticity meant that periodic reductions in population size < *K* (i.e., bottlenecks) unmasked deleterious mutations through increased inbreeding and genetic drift. Demo‐genetic feedback reduced mean individual fitness and population growth rate (Figure 3) and increased extinction probability by 0.24 and 0.48 compared to that observed in the demographic and genetic models, respectively (Figure 4). Base model choice also affected *T*_*ext*_ in simulated populations (Figure 4). On average, extinction occurred 1444 cycles (≈ years) sooner in simulations of the demo‐genetic base model compared to the demographic base model (Figure 4). We did not compare *T*_*ext*_ between the demo‐genetic and genetic base models because we arbitrarily aborted simulations of the genetic base model at 5000 cycles after demographic decline (at which time all iterations of the genetic base model remained extant).

We then calculated the time to extinction and the probability of extinction in simulations of the four genetic‐rescue scenarios and compared them to values obtained from the demo‐genetic base model. The time to extinction did not differ among the genetic rescue scenarios and the demo‐genetic base model. However, the simulated genetic rescue slightly reduced the extinction probability by 3%–9% relative to the demo‐genetic base model, with scenario 4 having the greatest impact (Figure 4).

### 4.3 Temporal Resolution of Simulation Cycles and Iterations

In this demonstration model, simulation ‘cycles’ approximate years, but this does not have to be the case. The duration represented by one cycle would depend on the system modelled and the study's aims; a cycle could represent periods < 1 year if, for example, seasonal variation in mortality is suspected to affect the demographic structure of a population. Cycles could also be > 1 year when, for example, the species exhibits low annual variation in mortality and breeds episodically (e.g., linked to multi‐annual environmental events). The time frame over which the outcomes of each simulation *iteration* occurs (i.e., time to extinction *T*_*ext*_ and probability of extinction at time *t* in the future *Pr*(*ext*); Figure 3 and Figure 4) depends strongly on the specific combination of parameter values used in a given model. The goal of the modelling exercise is not to predict the specific outcome of genetic rescue in terms of *T*_*ext*_ and *Pr*(*ext*), but rather to compare and rank different scenarios of genetic rescue based on their *relative influence* on *T*_*ext*_ and *Pr*(*ext*) in virtual populations (see *Using simulations to inform applied genetic rescue*).

Below, we discuss how demo‐genetic models can be developed and applied using available genetic data for populations of threatened species that are targeted for genetic rescue. We use a case study of threatened native Australian marsupials to demonstrate the types of genetic data that are available and suggest a set of modelling strategies and how data can be used to develop and apply models.

## 5 Australian Threatened Marsupials: A Case Study and Guide to Parameterize Models Using Genetic Data

### 5.1 Availability of Genetic Data for Threatened Australian Marsupials

Australia is one of the megadiverse nations characterized by its high endemism (e.g., 87% of its mammal fauna endemic) (Woinarski et al. 2015). Australia also has the worst record of mammal extinction of any nation—at least 17 out of its ~ 179 species of native marsupials have gone extinct in the last 200 years (Legge, Hayward et al. 2023a; Woinarski et al. 2015). In contrast, not one of the ~130 New World marsupial species has gone extinct over the same period (Martin et al. 2022). Marsupial extinctions have been driven mainly by novel predation pressure from introduced cats (*Felis catus silvestris*) and European red foxes ( *Vulpes vulpes* ), as well as from habitat loss, altered fire regimes, and climate change, the effects of which all interact (Legge, Rumpff et al. 2023b; Legge, Hayward et al. 2023a). Today, there are an additional 110 Australian marsupials (~ 65% of extant species) listed as threatened under the Australian *Environment Protection and Biodiversity Conservation Act 1999* (EPBC Act; total number comprises 55 Vulnerable, 46 Endangered, and 9 Critically Endangered species, subspecies or geographically defined populations). Many of these species now only occur in small (< 1000 individuals) and isolated populations occupying < 10% of their former geographic ranges (Legge, Hayward et al. 2023a; Woinarski and Fisher 2022). The combined effects of inbreeding and genetic drift are now recognized as additional threats to the persistence of many threatened marsupial species, making the prospect of genetic rescue a serious management consideration (Cowen et al. 2023; Farquharson et al. 2021; Hoffmann et al. 2021; Nilsson et al. 2023; Nistelberger et al. 2023).

Rapid advances in sequencing technology and bioinformatics have increased the feasibility of obtaining high‐resolution (and lower‐cost) genomic data for threatened species (Bernatchez et al. 2024; Hogg et al. 2022). These genomic data, as well as the many studies based on microsatellite DNA (e.g., DeWoody et al. 2021) can be leveraged to develop and apply simulation models of genetic rescue. In this section, we focus on Australian threatened marsupials to suggest approaches based on publicly available data to parameterize, calibrate, and validate demo‐genetic models. The aim is to guide decision‐making in the implementation of genetic rescue (cf. guiding decision‐making on the need for genetic rescue). We focus on marsupial species listed as Endangered and Critically Endangered under the federal (EPBC Act) 1999 as an exemplar of model‐based decision‐making in genetic rescue (described below under *Using simulations to inform applied genetic rescue*). Our approach can be applied to any species or population irrespective of threat status.

We searched *Web of Science* on 7 September 2023 for peer‐reviewed publications that provide estimates of population genetic/genomic measures of diversity and inbreeding in marsupials using the following four search terms: (*i*) marsupial* AND genetic* AND microsatellite*; (*ii*) marsupial* AND genomic* AND snp; (*iii*) marsupial* AND genomic* OR genetic* AND bottleneck; and (*iv*) marsupial* AND genomic* AND divers* OR demog*. Our searches returned 173 results, of which 135 were unique. We then subsetted the list to primary articles (i.e., not review articles) that included the names of native Australian marsupials listed as Endangered or Critically Endangered under the EPBC Act (Table S1). Next, we removed papers that were about the development of molecular resources (e.g., microsatellite development, reference genomes) and did not include estimates of population genetic diversity, structure, or inbreeding.

The procedure described above resulted in a sample of 73 articles that provided genetic data for 21 species. We subsequently repeated the same procedure on 19 November 2024 to update our results to include recently published articles. We also completed some additional opportunistic searches to find articles that we knew we had missed in our original searches. We applied the same pruning procedure described above to the resulting list of papers from updated searches. Our final sample resulting from all searches included 79 papers for 21 species (Table 4).

**TABLE 4:** Examples of published genetic data for Australian marsupials with which to develop, parameterize, calibrate, and validate simulation models of genetic rescue. The method of literature searching is described in the main text. Searches were comprehensive but not exhaustive; our intention was to highlight the range of different types of genetic data available for a diversity of threatened marsupials. Therefore, not all published literature on the genetics of Australian marsupials is shown in the summary. Species included in the table are listed as Endangered or Critically Endangered in the *Environment Protection and Biodiversity Conservation Act 1999* (EPBC Act), many of which are the focus of genetic management.

Species	Data types	Localities	Analyses	Diversity loss	References
*Bettongia penicillata*	Microsatellites, mtDNA, ancient DNA, SNPs	4+ pop, 9 localities	Diversity, gene flow, pop structure, phylogeny, ancient vs. modern diversity, pop viability modelling (microsatellite‐based), captive breeding design, inbreeding (SNP‐based)	Yes; ancient DNA comparisons	Farquharson et al. 2021; Pacioni et al. 2011, 2013, 2015, 2018, 2020
*Bettongia tropica*	Microsatellites	1 pop	Mating systems; relatedness and individual dispersal	Not directly reported	Pope et al. 2012
*Burramys parvus*	Microsatellites, mtDNA	13 localities, ~ 4 pop	Pop structure, diversity, *N*_e_, bottleneck, phylogeny	Signature of genetic bottleneck	Mitrovski et al. 2007; Weeks et al. 2016
*Dasyurus hallucatus*	Microsatellites, SNPs, mtDNA	10+ localities	Pop structure, diversity, *N*_e_ (temporal comparisons), selection	Yes, in island translocations vs mainland, and in historical vs contemporary *N*_*e*_	Cardoso et al. 2009; Hohnen et al. 2016; How et al. 2009; Spencer et al. 2017; von Takach et al. 2022, 2024; Weeks et al. 2016
*Dasyurus maculatus*	Microsatellite	12+ localities	Pop structure, diversity, historical phylogeography	Maybe	Firestone et al. 1999; Ruibal et al. 2009, 2010; Weeks et al. 2016
*Dasyurus viverrinus*	Microsatellites	9 localities	Pop structure, diversity, bottleneck, *N*_e_	Signature of genetic bottleneck	Cardoso et al. 2014
*Gymnobelideus leadbeateri*	Microsatellites, mtDNA, SNPs	4+ pop	Pop structure, diversity (including temporal trends), *N*_e_, inbreeding, inbreeding depression (inheritance of fitness traits), population viability simulations/genetic rescue recommendations	Yes; e.g., temporal declines in heterozygosity	(Hansen et al. 2009; Hansen and Taylor 2008; Zilko et al. 2020, 2021
*Isoodon obesulus*	Microsatellites	10+ localities	Pop structure, diversity, gene flow	Not directly, but pop structure inferred impacted by fragmentation	Li et al. 2013, 2014, 2015, 2016; Ramalho et al. 2018; Zenger et al. 2005; Zenger and Johnston 2001
*Lagorchestes hirsutus*	Microsatellites, mtDNA	3 pop	Pop structure, diversity, phylogeny	Not directly reported	Eldridge et al. 2004; Eldridge and Potter 2019
*Lasiorhinus krefftii*	Microsatellites	1 pop	individual relatedness and dispersal	Not directly reported	Taylor et al. 1997
*Myrmecobius fasciatus*	Microsatellites	1 pop	Parentage, diversity	Not directly reported	Spencer et al. 2020
*Onychogalea fraenata*	Microsatellites	Remnant pop, captive‐bred, translocated cohorts	Pop structure, diversity, parentage, reproductive success	Reduced diversity following translocation	Sigg 2006; Sigg et al. 2005
*Parantechinus apicalis*	Microsatellites, mtDNA	Mainland +3 island pop	Pop structure, diversity, bottlenecks, phylogeography, population viability analysis to guide translocations	Signatures of bottlenecks and *N*_e_ decline on islands	Aisya et al. 2022; Mills et al. 2004; Thavornkanlapachai et al. 2019, 2021
*Perameles* *gunnii*	Microsatellites, mtDNA	7+	Pop. structure, diversity, gene flow	Lower diversity in modern samples compared to historical samples	Black et al. 2024; Weeks et al. 2013
*Perameles bougainville*	Microsatellites, mtDNA	5 islands (both natural + introduced pop)	Pop structure, diversity, bottleneck tests	Not recently; long‐term low diversity followed by captive mixing = higher diversity	Smith and Hughes 2008; White et al. 2018
*Petauroides volans*	Microsatellites	11 sites	Pop structure and connectivity, parentage	weak evidence	Taylor et al. 2007
*Petrogale lateralis*	Microsatellites, mtDNA	4+ wild and various captive	Pop structure and dispersal, diversity, parentage, taxonomy, population viability analyses	Various findings, but evidence of recent mainland bottlenecks and long‐term low diversity on islands	Eldridge et al. 1999, 2004; Eldridge and Potter 2019; Nilsson et al. 2023; Potter et al. 2017; Ruykys and Lancaster 2015; West et al. 2018
*Phascolarctos cinereus*	Microsatellites, mtDNA, SNPs, MHC, WGS, transcriptome	91+ locs, 5 main clusters	Many and various	Signature of genetic bottleneck	Cheng et al. 2018; Cristescu et al. 2010; Hobbs et al. 2014; Johnson et al. 2018; Kjeldsen et al. 2019; Lott et al. 2022, 2024
*Potorous gilbertii*	Microsatellites, mtDNA	1 extant pop, plus captive	Pop structure, diversity, bottleneck tests, *N*_e_	Yes; signature of genetic bottleneck	Sinclair et al. 2002
*Sarcophilus harrisii*	Microsatellites, mtDNA, SNPs, MHC, WGS, transcriptome	10+ localities	Many and various, including population demography	Yes; genomic signatures of repeated bottlenecks	Brandies et al. 2019; Cheng et al. 2012; Cheng and Belov 2012; Cui et al. 2015; Deakin and Belov 2012; Farquharson et al. 2022; Grueber et al. 2021; Jones et al. 2003, 2004; Miller et al. 2011; Murchison et al. 2012; Silver et al. 2021; Stahlke et al. 2021; Ujvari et al. 2014; Woods et al. 2018; Wright et al. 2019
*Sminthopsis psammophila*	Microsatellites, mtDNA	11+ Sites, ~ 3 pop.	Pop structure, diversity, bottleneck tests, *N*_e_, relatedness	Signature of genetic bottleneck	McLean et al. 2014, 2018

Abbreviations: het, heterozygote; MHC, major histocompatibility complex; mtDNA, mitochondrial DNA; *N*_c_, census population size; *N*_e_, effective population size; pop, population(s); SNPs, single nucleotide polymorphisms; WGS, whole‐genome sequencing.

The type of genetic data available for the species on which we focused ranged from microsatellite markers to genome‐wide single nucleotide polymorphisms obtained from reduced‐representation sequencing (RRS; e.g., DArTseq, ddRAD) to whole genomes. Of the 21 species for which we found published genetic data, all had microsatellite data, 13 species had mitochondrial DNA (mtDNA), nine species had genome‐wide SNPs, and two species had whole‐genome sequences— *Phascolarctos cinereus* (koala) and *Sarcophilus harrisii* (Tasmanian devil). There was also one species in which ancient DNA had been sequenced— *Bettongia* *penicillata* (woylie). Most species (15) were represented by one or two types of sequence data, while fewer (6) had three or more data types. Species with the most genetic data included *Bettongia penicillata* , *Dasyurus hallucatus* (northern quoll), *Perameles bougainville* (western barred bandicoot), *Gymnobelideus leadbeateri* (Leadbeater's possum), *Phascolarctos cinereus* , and *Sarcophilus harrisii* .

### 5.2 Using Data in Model Development and Application

Here we link the literature review of genetic data in marsupials with the modelling sections above by discussing potential strategies for simulating genetic rescue. Genetic data can be used at different stages of model development and can be applied at one or all stages of parameterization, calibration, and/or validation. Given the flexibility of individual‐based modelling platforms and the wide range of context‐specific objectives and data types, we cannot be prescriptive about strategies for the use of data in model development and application. Instead, we provide a few examples of how data could be combined with genetically explicit, individual‐based models to simulate genetic rescue. The sensitivity of the model outcomes to parameter uncertainty can be quantified using global sensitivity analyses (Prowse et al. 2016) that have the dual benefit of quantifying uncertainty in model outcomes as well as prioritizing which data are essential for realistic model predictions. Quantitative evaluation of parameter values and their influence on simulated outcomes should avoid the misuse of Neyman‐Pearson Type I error estimates (*p* values) and instead focus on effect sizes (see White et al. 2018 for an explanation of why frequentist statistical hypothesis tests are inappropriate for interpreting simulation model results).

#### 5.2.1 Strategy 1: Simulate Genetic Rescue Using Virtual Sequence Variation

This strategy involves simulating *de novo* mutations that arise and change in frequency during a burn‐in period, followed by population decline before simulating genetic rescue. Here, parameter values need to be defined for the mechanisms that generate virtual sequence variation, including mutation rate(s), recombination rate(s), as well as the fitness effects and dominance of virtual *de novo* mutations. Because populations needing genetic rescue are unlikely to have the necessary data to estimate these mechanistic parameters, estimates can be used from (in order of preference) other populations of the same species, related species, or unrelated species to fill the gap. For example, a mutation rate for koalas has recently been estimated (T. Kovacs et al., in prep), which should supersede estimates for *Drosophila* or humans if simulating genetic rescue of a marsupial population. Similarly, in a mouse subspecies ( *Mus musculus castaneus* ), the distribution of fitness effects of nonsynonymous mutations is bimodal: most mutations are nearly neutral, and some mutations are strongly deleterious (Kousathanas and Keightley 2013). Parameters for which there are no data can be estimated from allometric relationships or based on other reasonable assumptions informed by theoretical predictions (e.g., Fisher's geometric model; Orr 2006). As stated above, cautious interpretation of model outputs should be guided by global sensitivity analyses to quantify and highlight uncertainty.

More commonly available data for target populations include sequence‐based estimates of genetic diversity and inbreeding (e.g., allelic richness, heterozygosity/homozygosity, inbreeding or related coefficients), and less commonly, genetic load. Such information could be used to calibrate (and ideally, validate) the mechanistic parameters in the model that give rise to virtual sequence variation. For example, rates of mutation and recombination, fitness effects, and dominance could be calibrated so that estimates of genetic parameters in simulated populations match empirical estimates derived from the target population. Known as ‘pattern‐oriented modelling’ this approach is widely used to calibrate and validate individual‐based models (Gallagher et al. 2021; Grimm et al. 2006). Once the genetic mechanisms that give rise to virtual sequence variation are calibrated, users could then run simulations of genetic rescue and compare them based on how much they affect virtual genetic diversity, inbreeding, or genetic load.

Pattern‐oriented calibration of mechanistic parameters should be guided by the type of sequence data available. For example, available data might include pre‐intervention estimates of genetic diversity obtained from microsatellites or genome‐wide single nucleotide polymorphisms. Empirical estimates of runs of homozygosity and genetic load might be obtained from the target population if whole‐genome sequencing data are available. Sequencing or genotyping genome‐wide single nucleotide polymorphisms is the most tractable way to capture important elements of genetic diversity without needing to define the adaptive role of functional genes in threatened populations (e.g., Kardos et al. 2021). Genome‐wide data might also describe regions of unknown adaptive function. Simulating how genetic rescue remasks realized genetic load and breaks up runs of homozygosity is an exciting area that will be increasingly valuable as the cost and analytical challenges associated with whole‐genome sequencing continue to decline. Similarly, simulations offer a powerful tool for guiding genetic rescue, targeting genetic diversity at specific coding regions, but this will be limited to systems in which the adaptive function of different variants has been well characterized (e.g., immunohistocompatibility complex genes for which particular alleles are known to improve resistance to infectious diseases). Whatever the type of data available for the target population, the aim should be to develop a model that produces sequence variation of a similar type and resolution to the data from which estimates of genetic diversity (or load) are derived in the target population.

#### 5.2.2 Strategy 2: Simulate Genetic Rescue Using Empirical Sequence Variation

Another strategy involves simulating genetic rescue of populations in which allele frequencies have been initialized from sequence (marker) data (‘empirical alleles’). Here, sequence data from the target population can be imported into the simulated populations (e.g., via a FASTA or VCF file) at a user‐defined time based on when the data were collected. For example, if data are collected at multiple time points during the decline of the target population, those from earlier sampling events could be imported into the simulation at a specific time, and the effect of simulated population dynamics on allele frequencies (or heterozygosity, genetic load, etc.) could be validated by comparing the simulated outcome to data from later sampling events. When data for the target population are only available from contemporary samples, they could be used to initialize allele frequencies at the point in the simulation when abundance in the virtual population matches that of the real population.

#### 5.2.3 Strategy 3: A Hybrid Approach

A third strategy combines elements of the two previous approaches by calibrating mechanistic parameters that mediate the influence of demo‐genetic feedback on virtual sequence variation *before* importing the empirical sequence data into the simulated population. Once the genetic mechanisms of demo‐genetic feedback are calibrated, users could run simulation burn‐ins without virtual alleles (to simulate historical equilibrium abundance followed by decline), and then sequence data from the target population could be imported into the simulated population at the appropriate time, such as when the simulated population size is similar to the abundance of the real population (if known) when the samples were collected.

## 6 Using Simulations to Inform Applied Genetic Rescue

### 6.1 Simulating Alternative Scenarios of Genetic Rescue

Here, we describe an approach to using simulations to compare and rank alternative potential scenarios of genetic rescue. Alternative scenarios facing decision‐makers might differ for a range of variables, for example: (*i*) population abundance (*N*) of the target and source populations (i.e., estimated census population size *N*_*c*_); (*ii*) effective population size (*N*_e_) of the target and source populations; (*iii*) carrying capacity (*K*) of the target population; (*iv*) mean heterozygosity of the target population; (*v*) genetic differentiation of the target and source populations; (vi) number of individuals translocated from the source population (translocated cohort) into the target population; (*vii*) number of translocation events; (*viii*) time between translocation events (frequency); (*ix*) sex ratio of the translocated cohort; and (*x*) age distribution (or stage‐structure) of the translocated cohort. In spatial simulations, additional variables could include: (*xi*) population density and dispersion of the target population, and (*xii*) probability of post‐release hyper‐dispersal of translocated individuals. This is not an exhaustive list of variables to compare genetic rescue scenarios, but they do include the most tractable ones to vary among alternative interventions.

The variables listed above can be altered to simulate alternative scenarios of genetic rescue, which can then be compared and ranked based on how they affect the outcome on simulated population dynamics and genetic composition. The ‘currency’ of the simulations is the probability of extinction *Pr(extinction)* at a specific time *t* in the future, and mean time to extinction (*T*_*ext*_) calculated for different genetic scenarios. We caution against using forward‐projection models for predicting specific outcomes of genetic rescue and population viability, which is not their intended purpose—forward‐projection models can be used to rank the relative success of virtual genetic‐rescue scenarios and prioritize real‐world strategies based on the relative probability of reducing inbreeding depression and increasing population viability and genetic diversity (Box 1).

BOX 1 **Step‐by‐step guide for developing and applying demo‐genetic simulations of genetic rescue in conservation.**

1. Define the objective of the genetic‐rescue intervention
  ○ Identify the target population and define the strategic objective (e.g., effect size and time frame for an increase in genetic diversity and improvement in per capita population growth).
2. Gather relevant data
  ○ Compile or collect demographic data (e.g., population size, survival and fertility, age structure, sex ratios)
  ○ Compile or collect genetic data (e.g., allele frequencies, inbreeding coefficients, heterozygosity, genetic load)
  ○ Include spatial data: habitat suitability, landscape permeability, and population distribution
3. Select and learn modelling software
  ○ Choose a suitable software platform based on the required capabilities (Table 3 for comparison of a representative set of programs)
  ○ Understand the software documentation and user interface or coding language (where applicable)
4. Develop the model framework
  ○ Define the base demographic model, including mechanisms for demographic stochasticity and density feedback
  ○ Add genetic processes (e.g., simulate mutation, recombination, drift, selection effects)
  ○ Link demographic and genetic processes to allow for demo‐genetic feedback
5. Parameterize the model
  ○ Use empirical data to set parameters for demographic and genetic processes
  ○ If empirical data are unavailable, use values from related species, theoretical predictions, or allometric relationships.
  ○ Do sensitivity analyses to identify critical parameters and quantify uncertainty
6. Simulate baseline and intervention scenarios
  ○ Run baseline simulations to establish control scenarios (e.g., no intervention)
  ○ Develop and simulate genetic rescue scenarios by varying factors such as translocation size, frequency, and source populations (see *Simulating alternative scenarios of genetic rescue* for a set of variables to consider)
  ○ Include spatial components if necessary, such as habitat connectivity or dispersal dynamics
7. Analyze simulation outputs
  ○ Compare outcomes of alternative scenarios using metrics such as probability of extinction, time to extinction, genetic diversity, and heterozygosity
  ○ Focus on effect sizes (rather than Neyman‐Pearson hypothesis tests) to rank scenarios and identify optimal interventions
8. Validate the model (if possible)
  ○ Compare simulation outputs to observed data from target or similar populations
  ○ Use pattern‐oriented modelling to refine parameters if discrepancies are detected
9. Interpret and apply results
  ○ Summarize findings in terms of relative scenario performance (i.e., change in extinction risk)
  ○ Provide clear recommendations for implementing genetic rescue (e.g., optimal cohort size, number of translocations)
10. Iterate and adapt
  ○ Update model with new data as they become available
  ○ Reassess scenarios to refine recommendations in response to changing conditions or additional insights

## 7 Conclusions

We have highlighted how demo‐genetic feedback can be incorporated into simulation models that test the relative benefits of genetic rescue. While theory predicts that demo‐genetic feedback plays an important role in extinction probability (i.e., driving populations to and keeping them in the extinction vortex) (Benson et al. 2016; Caughley 1994; Gilpin and Soulé 1986; Melbourne and Hastings 2008), and some empirical validation exists (Fagan and Holmes 2006; Willi and Hoffmann 2009), there are no published examples showing how demo‐genetic feedback influences the outcome of virtual genetic rescue. This lack of evidence is despite available software now possessing the capability to include demo‐genetic feedback in predictions of relative population viability.

There are several opportunities for and limitations to the development of simulation models of genetic rescue, and how they can be applied to inform conservation management. Some of those limitations arise due to the challenge of parameterizing models with empirical data, which in many instances will be challenging or impossible to collect (especially for threatened species). We suggest that choosing not to proceed with genetic rescue due to data limitations risks complacency and that the risks and benefits of mixing or not mixing need to be taken into consideration in the context of immediate threats to small (< 1000 censused individuals) populations. In other words, inaction is still a management decision potentially affecting the persistence of threatened species. In the case of missing data, we advocate instead the diligent use of models parameterized with allometric relationships, analogous species (e.g., congenerics), or other reasonable assumptions and the cautious interpretation of model outputs guided by global sensitivity analyses to quantify and highlight uncertainty.

Populations of threatened species are increasingly at risk of extinction from the mutual reinforcement of demographic and genetic processes eroded by habitat loss and degradation, invasive species, exploitation, climate change, and their synergistic interactions. Even if habitat protection and ecological restoration grow apace as needed, the genetic consequences of population bottlenecks could persist and undermine population recovery. Developing simulation models to inform genetic rescue has the potential to increase the confidence of conservation practitioners to implement the intervention and offer guidance on how to improve the probability of success while avoiding unintended negative consequences. Simulations also offer an exciting avenue for future research, with many outstanding questions remaining that warrant attention (Box 2).

BOX 2 **Outstanding questions.**

Models of genetic rescue should aim to answer the following questions:

1. How sensitive is extinction probability to the number of individuals translocated into the target population and the frequency of those translocations?
2. How long (on a scale of generations) does it take to observe an effect in terms of masking genetic load and increasing population viability of the target population? This is particularly relevant in the context of realistic management timelines and the suggestion of differentiating between short‐term (F_1_, F_2_) versus long‐term (F_3_) effects of genetic rescue (Hoffmann et al. 2021).
3. How intensively does the target population need to be sampled to estimate the effect of genetic rescue on observed heterozygosity and genetic diversity?
4. Does the spatial configuration of translocations make a discernible effect on the outcome of genetic rescue? These considerations might only be relevant under specific circumstances (e.g., species with low dispersal, small, fenced reserves or island sanctuaries).

## Ethics Statement

The research presented in the current manuscript meets the ethical guidelines for research in Australia. No animals were used in the research, and we did not require approval from an Animal Ethics Committee.

## Conflicts of Interest

The authors declare no conflicts of interest.

## Supporting information


Data S1.

## Data Availability

All *SLiM* code required to run the simulations is available at doi:10.5281/zenodo.10939288 github.com/cjabradshaw/demo‐genetic
