# Supplementary material for: A Guide for Developing Demo‐Genetic Models to Simulate Genetic Rescue
Source: Evol Appl. 2025 May 14;18(5):e70092. doi: 10.1111/eva.70092 (PMC12076008; doi:10.1111/eva.70092)
Supplement: Supplementary file 1 — Data S1. [file EVA-18-e70092-s001.docx]

**SUPPLEMENTARY MATERIAL**

**Development of toy model**

Our goal was to demonstrate that the mutual reinforcement of demographic and genetic processes mediate extinction dynamics in small (virtual) populations. The implication is that the development of models for simulating genetic rescue should incorporate mechanisms that permit the emergence of demo-genetic feedback. We started by developing a set of three base models in which simulated population dynamics emerge from demographic stochasticity only (demographic model), genetic drift and inbreeding only (genetic model), and the mutual reinforcement of demographic stochasticity, genetic drift, and inbreeding (demo-genetic model). In developing these separate base models, we are not suggesting that demographic and genetic processes operate independently in natural populations. Rather, we take advantage of the ability to ‘turn on and off’ particular mechanisms in the simulations to examine their influence on population persistence and genetic diversity. In other words, our goal was to demonstrate that demographic and genetic processes are not independent and models that include both types of processes are more realistic. Using our toy model to demonstrate this point, we built three base models which varied specifically in the sources of among-individual variance in survival probability. We describe the key differences and similarities among each of the three base models below. We then explain in more detail how each model was parameterised on the sections that follow.

1. Demographic model — in this model, virtual *de novo* mutations were strictly neutral (i.e. no deleterious or beneficial effects of mutations on survival probability), but random variance in survival probability increased with decreasing abundance. We use the term ‘demographic model’ to represent the inclusion of mechanisms underlying the increased influence of demographic stochasticity on depensation as populations decline.

2. Genetic model — in this model, the effects of virtual *de novo* mutations on individual survival probability could either be neutral or deleterious (not beneficial), but the variance in survival probability did not increase with decreasing abundance. We use the term ‘genetic model’ to represent the inclusion of mechanisms that allow genetic drift and inbreeding in small populations to cause inbreeding depression and unmasking of genetic load.

3. Demo-genetic model — in this model, all the mechanisms and processes described above were incorporated simultaneously and caused the emergence of the mutual reinforcement of demographic and genetic mechanisms of depensation.

The basic elements that remained constant across all three models were: (*i*) individuals reproduced sexually, (*ii*) individuals started reproducing at 3 years old and had the opportunity to reproduce every year up until a maximum life span of 12 years, and (*iii*) variation in fitness manifested via variation in survival, but not fertility. This is the default specification in non-Wright-Fisher models in *SLiM*, but this assumption could be relaxed to include among-individual variation in survival and fertility.

*Demographic model*

This model included only the influence of demographic stochasticity on population growth. Here, we set the *variance* in survival probability to increase as population size declined to model the increased influence of (component) demographic stochasticity on (ensemble) per-capita population growth rate. Because we are using a non-Wright Fisher model in *SLiM*, individual fitness was determined by survival probability (viability selection) rather than fertility, although variation in fertility could be implemented in future models. To introduce stochastic variation in survival, we multiplied individual survival probability by a weighting factor drawn from a *β* distribution (also bound to 0 and 1), which randomly modified individual survival. We varied the shape of the *β* distribution (from which survival weights were drawn) as a function of population size (Fig. S1). The shape of *β* distributions are defined by two parameters, 𝛼 and 𝛽. Importantly, we defined the way combinations of 𝛼 and 𝛽 varied with population size to ensure there was no correlation between the mean and variance of the survival weighting factor. We did this to ensure that the impact of demographic stochasticity on population growth was due to increases in variance in survival probability rather than decreases in mean fitness. The demographic model did not include deleterious or beneficial mutations, such that there were no mutational effects on individual fitness and population growth. Neutral mutations were included to observe the effect of drift during runs of the simulation.

Genetic model

This model included only neutral and partially recessive deleterious mutations (no beneficial mutations) and did not include demographic stochasticity. The fitness effects of partially recessive deleterious mutations were drawn from a *γ* distribution with shape parameters -0.03 and 0.2. Most mutation effects were weakly to moderately deleterious, with rarer mutations of larger effect. A dominance coefficient = 0.1 made them almost recessive (i.e., incomplete dominance; Glossary main text). Partially recessive deleterious mutations spontaneously arose in the population during simulation burn-in. These mutations then had the potential to increase in frequency and become exposed in the homozygous state through genetic drift and inbreeding, causing reductions in individual fitness (survival probability) in homozygous individuals (inbreeding depression) and reducing population mean fitness (realised genetic load).

Demo-genetic model

This model included both demographic stochasticity and partially recessive deleterious mutations as described. Integrating stochastic demographic and genetic processes into the model allowed their mutual reinforcement (via density feedback) to emerge and influence population dynamics.


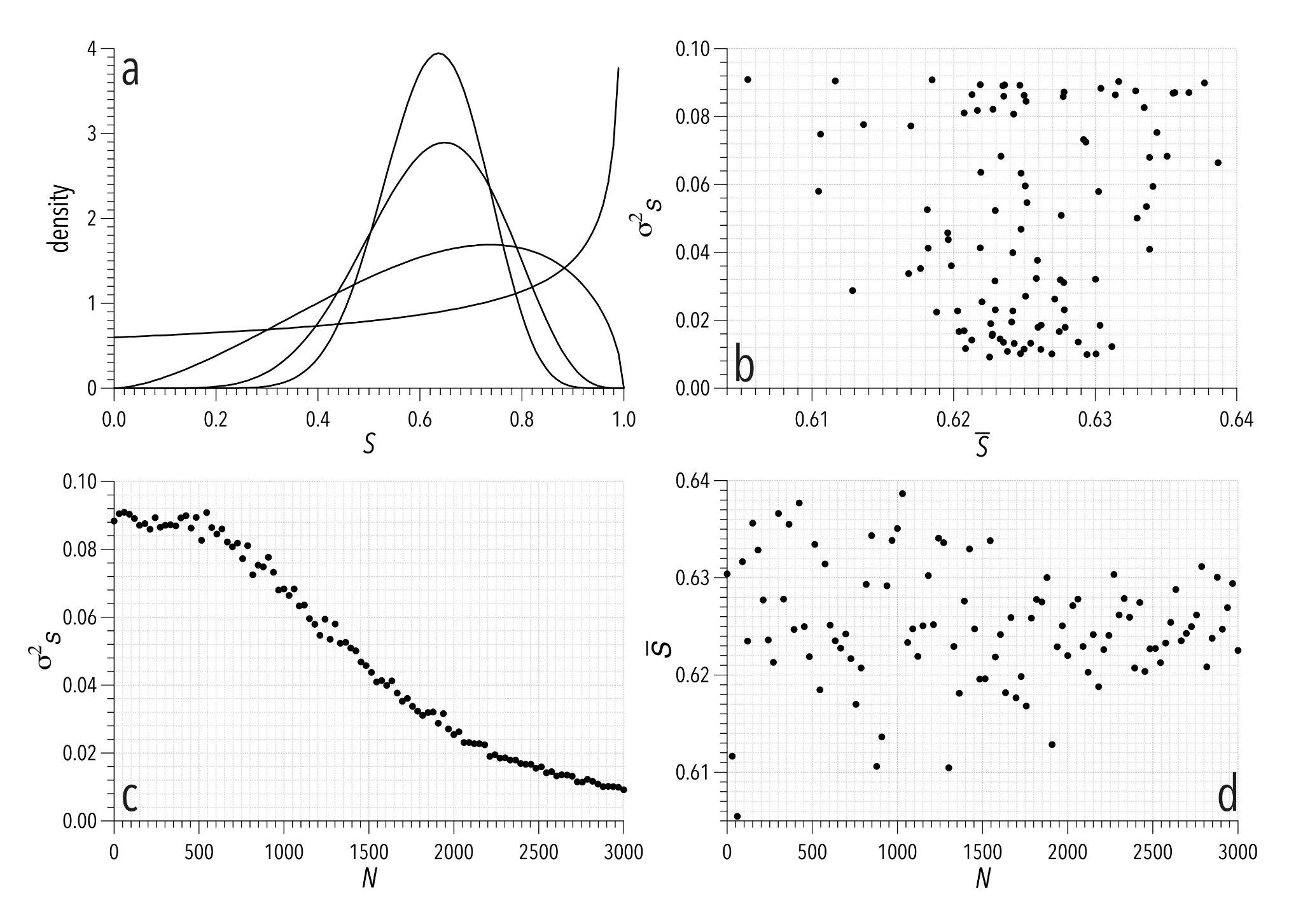


###### **Figure S1. Parameterising demographic stochasticity.** We parameterised β distributions of survival probability weighting factors with different 𝛼 and 𝛽 parameters. The parameters varied as a function of population abundance so that the shape of the probability distributions (a) had highest density at mean survival probability and low variance when populations were large (shown by the tallest and narrowest distribution), and became

###### progressively more spread out around the mean (i.e., increasing variance in survival probability) with decreasing abundance. Importantly, changes in

###### the shape of the β distributions intentionally avoided a (b) correlation between mean survival probability ($\underline{S}$) and abundance (N), but did induce an (c) increase in variance in survival probability ($\sigma_{S}^{2}$) with decreasing N. Consequently, there was (d) no relationship between mean and variance in survival probability. R code to reproduce this figure available at github.com/cjabradshaw/demo-genetic.

**Table S1. Australian *Environment Protection and Biodiversity Conservation* (EPBC) *Act*-listed Endangered and Critically Endangered marsupials.** For some species, EPBC listing only applies to particular subspecies or geographically defined populations (indicated in brackets in the second column). List current as of 21 November 2024.

| **Scientific species name** | | **English name (listed populations)** | **Indigenous language name(s)** |
| --- | --- | --- | --- |
| Critically Endangered | | | |
| 1 | *Gymnobelideus leadbeateri* | Leadbeater's possum |  |
| 2 | *Lasiorhinus krefftii* | northern hairy-nosed wombat | yaminon |
| 3 | *Petrogale concinna concinna* |  | nabarlek (Victoria River District) |
| 4 | *Potorous gilbertii* | Gilbert’s potoroo | ngilkat |
| 5 | *Pseudocheirus occidentalis* | western ringtail possum | ngwayir, womp, woder, ngoor, ngoolangit |
| Endangered | | | |
| 1 | *Antechinus argentus* | silver-headed *Antechinus* |  |
| 2 | *Antechinus arktos* | black-tailed *Antechinus* |  |
| 3 | *Bettongia penicillata ogilbyi* | woylie |  |
| 4 | *Bettongia tropica* | northern bettong |  |
| 5 | *Burramys parvus* | mountain pygmy-possum |  |
| 6 | *Dasyuroides byrnei* | kowari | kariri [Diyari] |
| 7 | *Dasyurus hallucatus* | northern quoll | digul [Gogo-Yimidir], wijingadda [Dambimangari], wiminji [Martu] |
| 8 | *Dasyurus maculatus gracilis* | spotted-tailed quoll (north Queensland) | yarri, burrumbil |
| 9 | *Dasyurus maculatus maculatus* | spotted-tail quoll / tiger quoll (SE mainland) |  |
| 10 | *Dasyurus viverrinus* | eastern quoll | luaner |
| 11 | *Isoodon obesulus obesulus* | southern brown bandicoot (east, south-east) |  |
| 12 | *Lagorchestes hirsutus* | rufous hare-wallaby (central Australia) | Mala [Warlpiri][Pintupi] |
| 13 | *Myrmecobius fasciatus* | numbat |  |
| 14 | *Onychogalea fraenata* | bridled nail-tail wallaby |  |
| 15 | *Parantechinus apicalis* | dibbler |  |
| 16 | *Perameles bougainville bougainville = Perameles bougainville* | western barred bandicoot (Shark Bay) |  |
| 17 | *Perameles gunnii* | eastern barred bandicoot (mainland) |  |
| 18 | *Petauroides volans* | greater glider (southern & central) |  |
| 19 | *Petaurus australis* | yellow-bellied glider (Wet Tropics) |  |
| 20 | *Petaurus gracilis* | mahogany glider |  |
| 21 | *Petrogale coenensis* | Cape York rock-wallaby |  |
| 22 | *Petrogale concinna canescens* | nabarlek (Top End) |  |
| 23 | *Petrogale concinna monastria* | nabarlek (Kimberley) |  |
| 24 | *Petrogale lateralis kimberleyensis* | west Kimberley rock-wallaby | wiliji |
| 25 | *Petrogale lateralis lateralis* | black-footed rock wallaby | warru [Aṉangu Pitjantjatjara Yankunytjatjara], moororong [nyungar] |
| 26 | *Petrogale persephone* | Proserpine rock-wallaby |  |
| 27 | *Phascolarctos cinereus* | koala (Combined QLD, NSW, ACT) |  |
| 28 | *Potorous longipes* | long-footed potoroo |  |
| 29 | *Sarcophilus harrisii* | Tasmanian devil |  |
| 30 | *Sminthopsis griseoventer aitkeni =*  *Sminthopsis fuliginosa aitkeni* | Kangaroo Island dunnart |  |
| 31 | *Sminthopsis psammophila* | sandhill dunnart |  |
